# The Cincinnati Meeting of the American Medical Association

**Published:** 1888-06

**Authors:** 


### *THE CINCINNATI MEETING OF THE AMERICAN MEDICAL ASSOCIATION.*

This seems to be the period of the rennaissance of the American Medical Association, if we may judge by the record of its recent meeting in Cincinnati; there appeared to be an especial grouping of favorable conditions to make this one of the best of all the many good annual gatherings of this honorable and popular organization.

In the first place, the city chosen was a favorable one—central, easily accessible from all parts of the country, with ample hotel accommodations, and a commodious building admirably adapted to the uses of such a convention.

Then, in choosing Dr. Dawson as chairman of the committee of arrangements, the Association was wise, for it is doubtful if this onerous post was ever filled by a more faithful or competent man. The registration system was simply perfect, and all the many other details for the convenience and comfort of the members showed the hand of a master at the helm.

The social features of this meeting were exceptionally pleasant in character. The departure from the stereotyped plan of private receptions, and the substitution therefor of the informal receptions at the Burnet House and at the Art Museum, together with the musical treat afforded by the fine concert at Music Hall, were delightful variations, pleasing to both eye and ear. Cincinnati is rapidly taking position as the first American city in music and art, and wisely afforded the visitors an opportunity to judge of her claims to this distinction.

If we turn to the scientific side of the convention, an equally favorable improvement may be noted. The chairmen of the sections this year delivered their addresses in their respective sub-divisions, where they properly belong, instead of in the general morning sessions, as heretofore.

The address on "General Medicine," by Dr. Roberts Bartholow, on Wednesday; that on "General Surgery," by Dr. E. M. Moore, on Thursday, and the one on "State Medicine," by Dr. H. P. Walcott, on Friday, all before the general morning sessions, were comprehensive, able, and instructive expositions of these several important departments of medical science.

The sections were, as a rule, well attended and the discussions unusually interesting, especially in the sections on surgery and obstetrics.

Dr. Senn's experiments with hydrogen gas in the diagnosis of intestinal wounds, were a contribution to the subject, of a high order of merit; and Thursday's discussion in the obstetric section on abdominal surgery was a scene well-nigh dramatic.

The practice, which has prevailed for the past three years, of the sections electing their respective chairmen, is to be commended as affording a better opportunity of selecting men for these important places who have done efficient work, than when the nominating committee performed this duty.

So long as the Association pursues the policy that prevailed in the meeting of 1888, of attending to its legitimate duties and avoiding acrimonious debates on codes and other mischievous petty questions, it will merit and receive the confidence and support of the better class of the profession; and, moreover, it need have no fear of opposition from any other organization, for its field is peculiarly its own, and it cannot be driven from it unless by its own suicidal acts.

---

In electing Dr. Wm. H. Wathen, of Louisville, Ky., to be Chairman of the Section on Obstetrics and Diseases of Women, and Dr. N. P. Dandridge, of Cincinnati, O., to be Chairman of the Section on Surgery and Anatomy, the American Medical Association has performed graceful acts of recognition to men who have been conspicuous for the scientific work they have accomplished in their respective sections. When a medical society brings such men to the front, it does itself honor.

